# Supplementary material for: Hypoxia induces HIF1α-dependent epigenetic vulnerability in triple negative breast cancer to confer immune effector dysfunction and resistance to anti-PD-1 immunotherapy
Source: Nat Commun. 2022 Jul 15;13:4118. doi: 10.1038/s41467-022-31764-9 (PMC9287350; doi:10.1038/s41467-022-31764-9)
Supplement: Supplementary file 1 — Supplementary Information [file 41467_2022_31764_MOESM1_ESM.pdf]

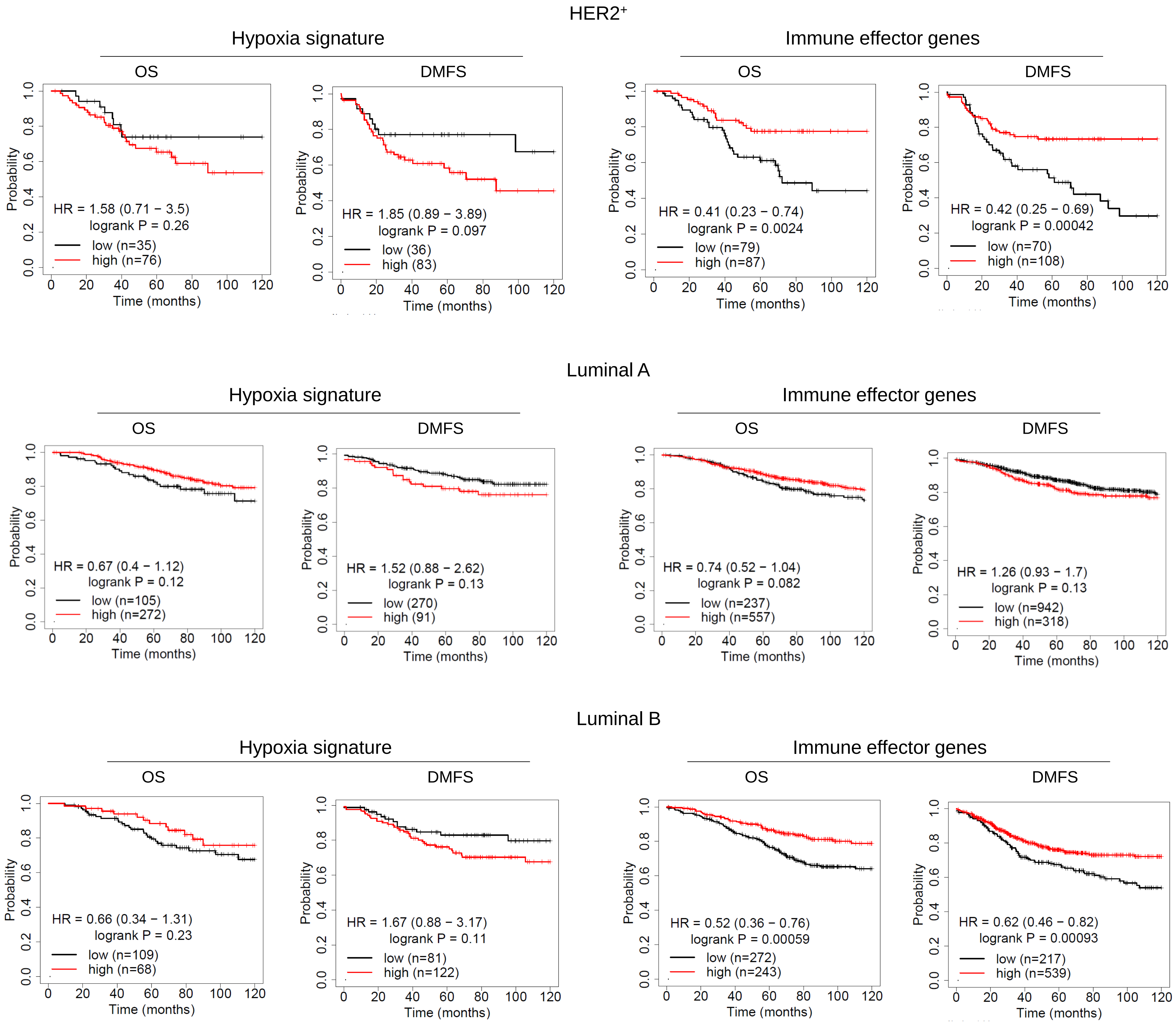

Supplementary Figure 1. Prognosis of hypoxic signature and immune effector molecules in HER2+ and Luminal breast cancer patients. Kaplan-Meier overall survival (OS) and distant metastasis-free survival (DMFS) analysis of the indicated gene signatures in HER2^{+}, Luminal A and Luminal B breast cancer patients. The number of subjects (n) is indicated in the graph. Analysis was conducted using the database from KM Plotter (https://kmplot.com/analysis/). Patients were splitted into low and high groups using the auto best–cut off of KM Plotter. The publicly available data are available in the KM-Plotter-Breast Cancer [https://kmplot.com/analysis/index.php?p=service&cancer=breast].

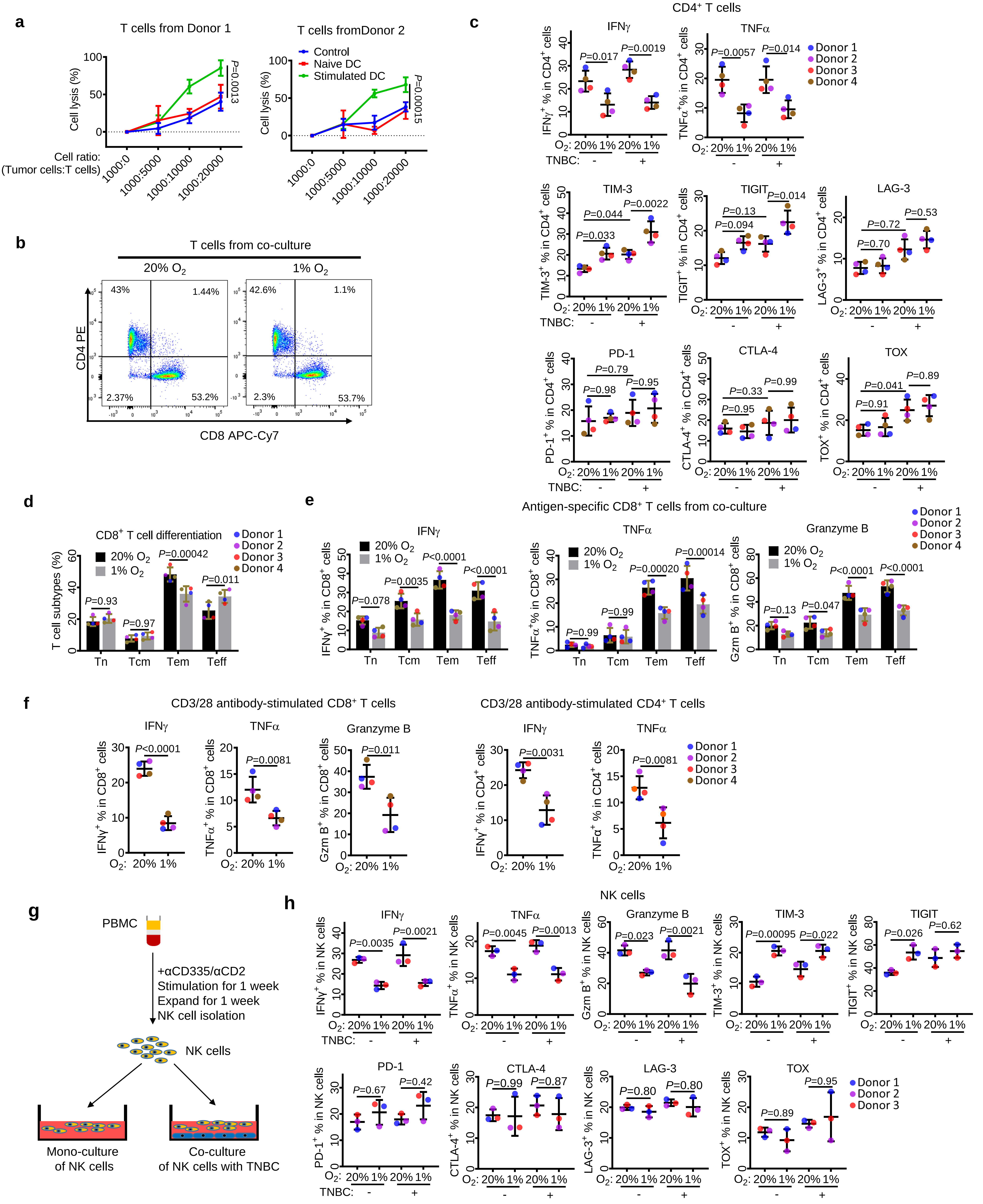

a
T cells from Donor 1
T cells fromDonor 2
Control
Naive DC
Stimulated DC
Cell lysis (%)
P=0.0013
P=0.00015
Cell ratio:
(Tumor cells:T cells)
1000:0
1000:5000
1000:10000
1000:20000
b
T cells from co-culture
20% O2
1% O2
43%
1.44%
2.37%
53.2%
42.6%
1.1%
2.3%
53.7%
CD4 PE
CD8 APC-Cy7
c
CD4+ T cells
IFNγ
TNFα
TIM-3
TIGIT
LAG-3
PD-1
CTLA-4
TOX
Donor 1
Donor 2
Donor 3
Donor 4
P=0.017
P=0.0019
P=0.0057
P=0.014
P=0.044
P=0.0022
P=0.033
P=0.13
P=0.094
P=0.72
P=0.70
P=0.53
P=0.79
P=0.98
P=0.95
P=0.33
P=0.99
P=0.041
P=0.91
P=0.89
O2: 20% 1% 20% 1%
TNBC: - +
d
CD8+ T cell differentiation
T cell subtypes (%)
20% O2
1% O2
P=0.93
P=0.97
P=0.00042
P=0.011
Tn
Tcm
Tem
Teff
e
Antigen-specific CD8+ T cells from co-culture
IFNγ
TNFα
Granzyme B
P=0.078
P=0.0035
P<0.0001
P=0.99
P=0.00020
P=0.00014
P=0.13
P=0.047
f
CD3/28 antibody-stimulated CD8+ T cells
CD3/28 antibody-stimulated CD4+ T cells
P<0.0001
P=0.0081
P=0.011
P=0.0031
g
PBMC
+αCD335/αCD2
Stimulation for 1 week
Expand for 1 week
NK cell isolation
NK cells
Mono-culture
of NK cells
Co-culture
of NK cells with TNBC
h
NK cells
P=0.0035
P=0.0021
P=0.0045
P=0.0013
P=0.023
P=0.00095
P=0.022
P=0.026
P=0.62
P=0.67
P=0.42
P=0.87
P=0.80
P=0.89
P=0.95

Supplementary Figure 2. Hypoxia induces dysfunction of human T and NK cells. a Cell lysis of TNBC cells cocultured with human T cells. Human T cells were treated with naïve DC cells (Naïve DC), TNBC cell lysate-primed DC cells (stimulated DC) or PBS (Control). Data are presented as mean ± SD of three independent experiments (n = 3). *P* values were determined by two-way ANOVA. **b** Representative flow cytograms of $CD4^+$ and $CD8^+$ percentage in human pan-T cells cocultured with human TNBC. **c** Flow cytometric quantifications of immune effector molecules and exhaustion markers level in $CD4^+$ T cells gated from human pan-T cells culture under the indicated conditions (n = 4 for each group). **d** Flow cytometric quantifications of differentiated CD8+ T cells subtype: Tn (naïve T cells), Tcm (central memory T cells), Tem (effector memory T cells), Teff (effector T cells), gated from human pan-T cell culture (n = 4 for each group). **e** Flow cytometric quantifications of immune effector molecules in different CD8+ T cell subtypes, gated from human pan-T cells culture under the indicated conditions (n = 4 for each group). **f** Flow cytometric quantification of immune effector molecules in CD8+ and CD4+ T cells gated from human pan-T cells culture under the indicated conditions (n = 4 for each group). **g** Schematic graph to demonstrate the expansion of human NK cells and coculture of human NK cells with human TNBC cells. **h** Flow cytometric quantifications of immune effector molecules and exhaustion markers level in human NK cells cultured under the indicated conditions (n = 3 for each group). For flow cytometric quantification, data are presented as mean ± SD of samples from 3-4 donors. *P* values were determined by one-way (**c** and **h**) or two-way (**d** and **e**) ANOVA with Turkey's test, or paired two-tailed t-test (**f**). Source data are provided as a Source Data file.

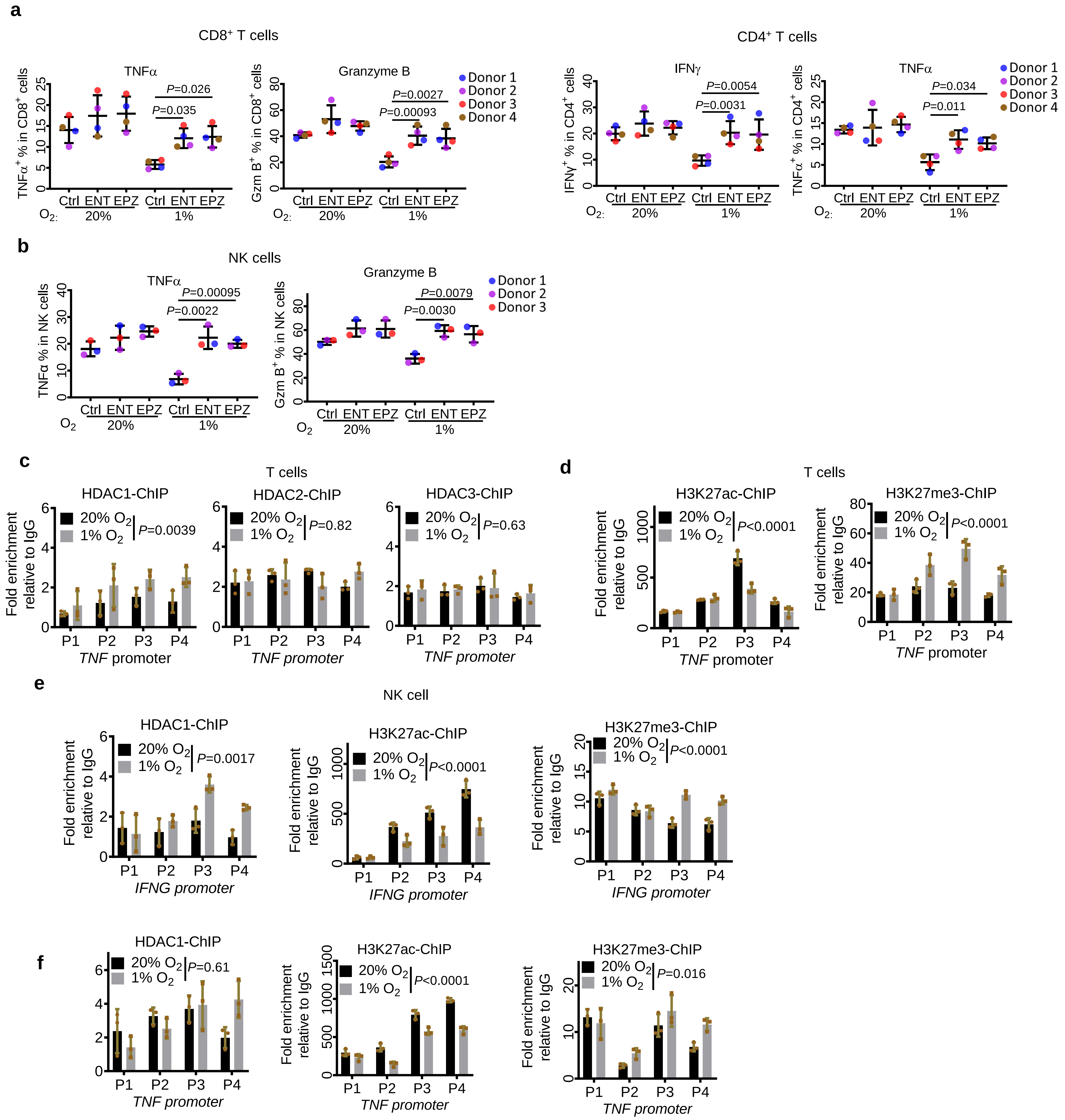

Supplementary Figure 3. Inhibitors of EZH2 and HDACs reverse dysfunction of T and NK cells under hypoxia. a-b Flow cytometric quantifications of immune effector molecules in CD8+ T cells and CD4+ T cells (**a**) and NK cells (**b**) cultured under indicated conditions. Data of **a** and **b** are presented as the mean ± SD of samples from different donors. N = 4 for T cells; n = 3 for NK cells. *P* values were determined by one-way ANOVA with Turkey's test. **c** ChIP-qPCR analysis of HDAC1, HDAC2 and HDAC3 occupancy on *TNF* promoter in human T cells. Four primers were designed to span the promoters of *TNF*, with P1 at -1300 to -1206b, P2 at -891 to -677b, P3 at -14 to +63b, P4 at +615 to +697b, relative to TSS. **d** ChIP-qPCR analysis of H3K27ac and H3K27me3 enrichment on *TNF* promoter in human T cells. **e-f** ChIP-qPCR analysis of HDAC1, H3K27ac and H3K27me3 enrichment on *IFNG* (**e**) and *TNF* (**f**) promoters in human NK cells. All ChIP-qPCR data (**c**, **d**, **e** and **f)** are presented as fold enrichment relative to IgG and expressed as mean ± SD of technical triplicates, representative of two independent experiments (n = 2). *P* values were determined by two-way ANOVA analysis. Source data are provided as a Source Data file.

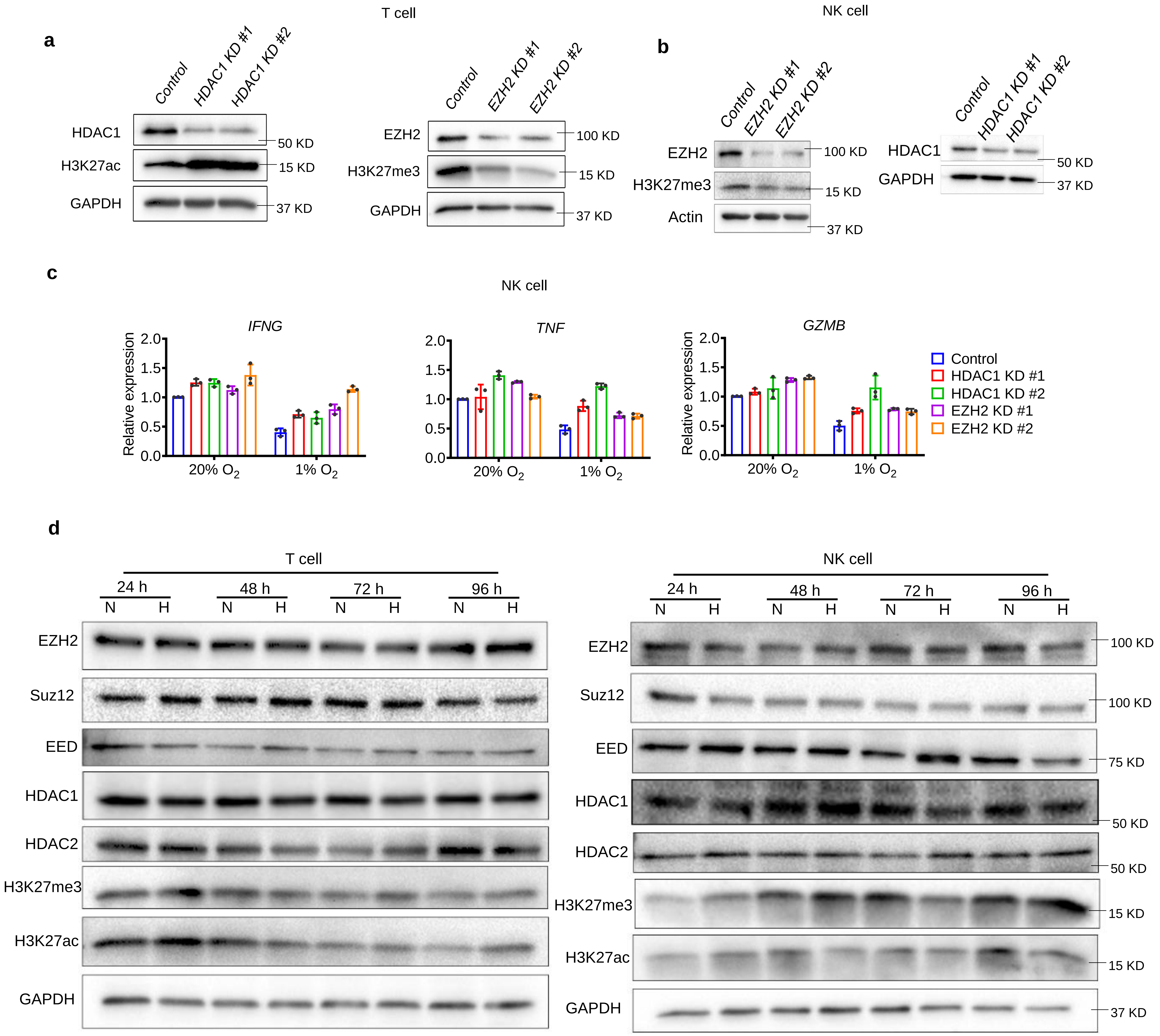

Supplementary Figure 4. Genetically knockdown of EZH2 and HDACs rescues immune effector molecules level in T and NK cells under hypoxia. **a-b** Representative western blot images (n = 2) to demonstrate knockdown of indicated genes in human T cells (**a**) and human NK cells (**b**). **c** RT-qPCR analysis assessing expression of *IFNG*, *TNF* and *GZMB* in human NK cells with indicated treatments. Data are presented as the fold change of mRNA level normalized to control group under normoxia, mean ± SD of technical triplicates, representative of two independent experiments (n = 2). **d** Western blot analysis of human T cells (left panel) and NK cells (right panel) cultured under hypoxia for different time windows. Images were representatives of two independent experiments (n = 2). Source data are provided as a Source Data file.

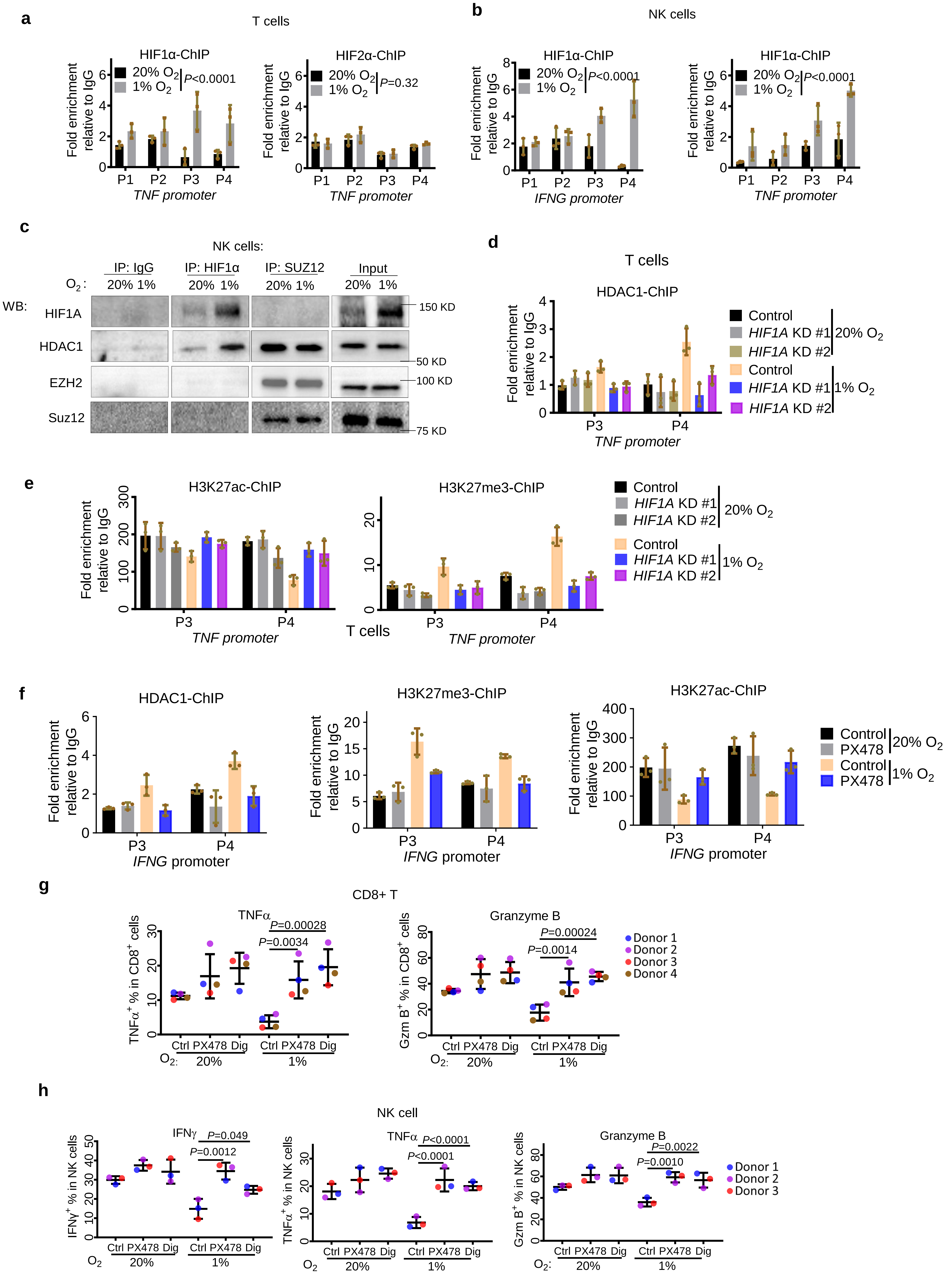
a
T cells
HIF1α-ChIP
HIF2α-ChIP
20% O2
1% O2
P<0.0001
P=0.32
Fold enrichment relative to IgG
P1 P2 P3 P4
TNF promoter
b
NK cells
HIF1α-ChIP
P<0.0001
IFNG promoter
TNF promoter
c
NK cells:
IP: IgG
IP: HIF1α
IP: SUZ12
Input
O2:
20% 1%
WB:
HIF1A
HDAC1
EZH2
Suz12
150 KD
50 KD
100 KD
75 KD
d
T cells
HDAC1-ChIP
Control
HIF1A KD #1
HIF1A KD #2
20% O2
1% O2
P3 P4
TNF promoter
e
H3K27ac-ChIP
H3K27me3-ChIP
T cells
f
HDAC1-ChIP
H3K27me3-ChIP
H3K27ac-ChIP
Control
PX478
IFNG promoter
g
CD8+ T
TNFα
Granzyme B
P=0.00028
P=0.0034
P=0.00024
P=0.0014
TNFα+ % in CD8+ cells
Gzm B+ % in CD8+ cells
Ctrl PX478 Dig Ctrl PX478 Dig
O2: 20% 1%
Donor 1
Donor 2
Donor 3
Donor 4
h
NK cell
IFNγ
P=0.049
P=0.0012
TNFα
P<0.0001
P<0.0001
Granzyme B
P=0.0022
P=0.0010
IFNγ+ % in NK cells
TNFα+ % in NK cells
Gzm B+ % in NK cells

Supplementary Figure 5. Depletion of HIF1α impairs hypoxia-induced epigenetic modification and rescues immune effector molecules expression in human T and NK cells. **a** ChIP-qPCR analysis of HIF1α and HIF2α occupancy on *TNF* promoter in human T cells. **b** ChIP-qPCR analysis of HIF1α occupancy on *IFNG* and *TNF* promoter in human NK cells. **c** Co-immunoprecipitation showing the physical interaction between HDAC1 and HIF1α and the interaction between HDAC1 and SUZ12 in human NK cells. Data is representative of two independent experiments (n = 2). **d** ChIP-qPCR analysis of HDAC1 occupancy on *IFNG* promoter in human T cells with *HIF1A* knockdown. **e** ChIP-qPCR analysis of H3K27ac and H3K27me3 on *IFNG* promoter in human T cells with *HIF1A* knockdown. **f** ChIP-qPCR analysis of HDAC1, H3K27ac and H3K27me3 enrichment on *IFNG* promoter in human NK cells with indicated treatments. All ChIP-qPCR data (**a**, **b**, **d**, **e** and **f)** are presented as fold enrichment relative to IgG and expressed as mean ± SD of technical triplicates, representative of two independent experiments (n=2). For ChIP data in **a** and **b**, *p* values were determined by two-way ANOVA analysis. **g** Flow cytometric quantifications of immune effector molecules in $CD8^+$ T cells gated from human pan-T cells cultured under the indicated conditions. Data are presented as the mean ± SD of samples from three donors (n = 3). *P* values were determined by two-way ANOVA with Turkey's test. **h** Flow cytometric quantifications of immune effector molecules in human NK cells cultured under the indicated conditions. Data are presented as the mean ± SD of samples from three donors (n = 3). *P* values were determined by two-way ANOVA with Turkey's test. Source data are provided as a Source Data file.

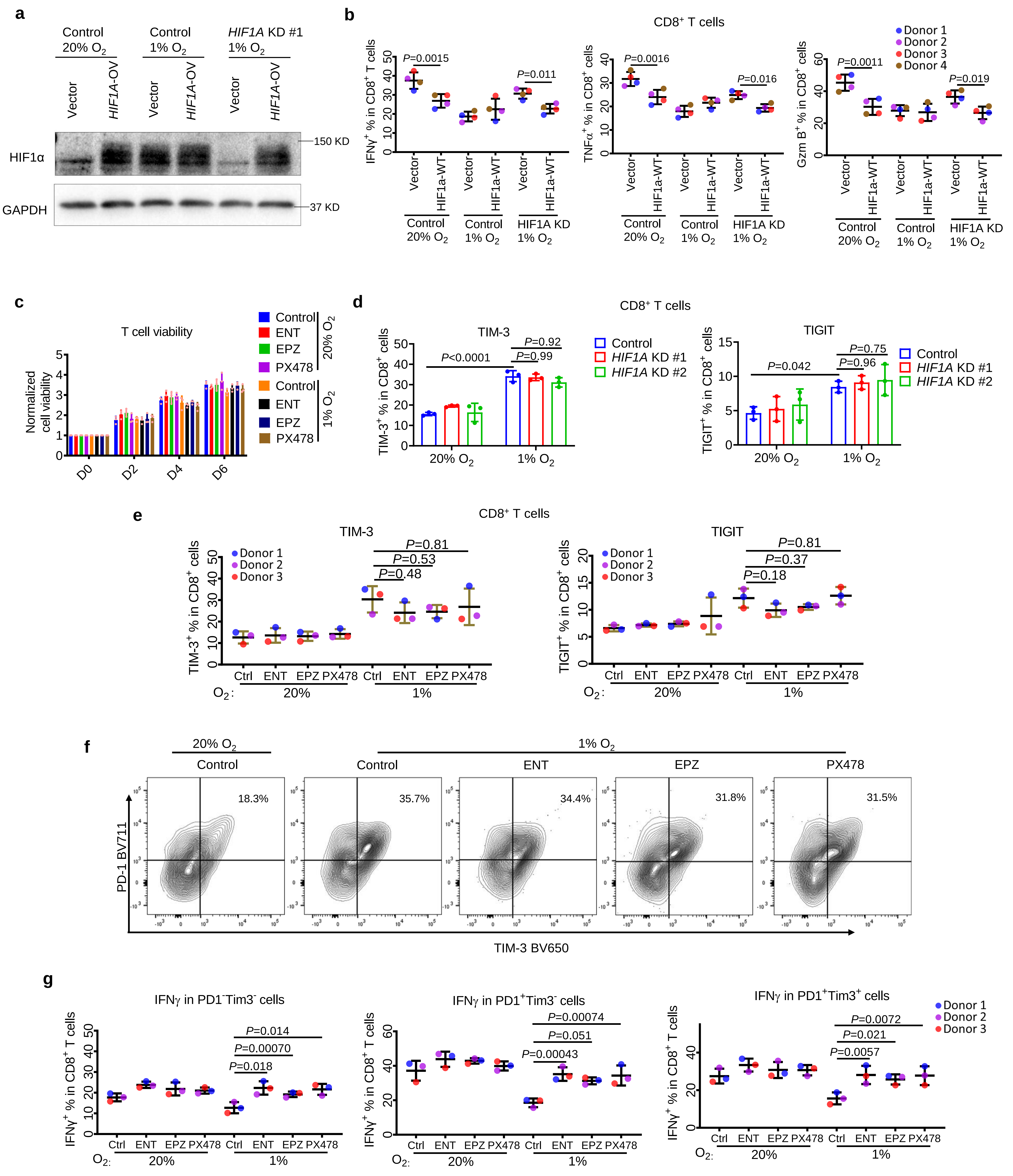

a
Control 20% O2
Control 1% O2
HIF1A KD #1 1% O2
Vector
HIF1A-OV
HIF1α
GAPDH
150 KD
37 KD
b
CD8+ T cells
IFNγ+ % in CD8+ T cells
TNFα+ % in CD8+ cells
Gzm B+ % in CD8+ cells
P=0.0015
P=0.011
P=0.0016
P=0.016
P=0.0011
P=0.019
Donor 1
Donor 2
Donor 3
Donor 4
Vector
HIF1a-WT
Control 20% O2
Control 1% O2
HIF1A KD 1% O2
c
T cell viability
Normalized cell viability
Control
ENT
EPZ
PX478
20% O2
1% O2
D0
D2
D4
D6
d
CD8+ T cells
TIM-3
TIM-3+ % in CD8+ cells
P<0.0001
P=0.92
P=0.99
TIGIT
TIGIT+ % in CD8+ cells
P=0.042
P=0.75
P=0.96
Control
HIF1A KD #1
HIF1A KD #2
20% O2
1% O2
e
CD8+ T cells
TIM-3
TIM-3+ % in CD8+ cells
P=0.81
P=0.53
P=0.48
TIGIT
TIGIT+ % in CD8+ cells
P=0.81
P=0.37
P=0.18
Donor 1
Donor 2
Donor 3
Ctrl ENT EPZ PX478 Ctrl ENT EPZ PX478
O2: 20% 1%
f
20% O2
1% O2
Control
Control
ENT
EPZ
PX478
18.3%
35.7%
34.4%
31.8%
31.5%
PD-1 BV711
TIM-3 BV650
g
IFNγ in PD1-Tim3- cells
IFNγ in PD1+Tim3- cells
IFNγ in PD1+Tim3+ cells
IFNγ+ % in CD8+ T cells
P=0.014
P=0.00070
P=0.018
P=0.00074
P=0.051
P=0.00043
P=0.0072
P=0.021
P=0.0057
Donor 1
Donor 2
Donor 3
Ctrl ENT EPZ PX478 Ctrl ENT EPZ PX478
O2: 20% 1%

Supplementary Figure 6. Targeting HIF1α, HDAC1, or EZH2 does not affect hypoxia-induced TIM-3 expression. **a** Representative western blot images (n = 2) to demonstrate overexpression of *HIF1A* in human T cells. **b** Flow cytometric quantifications of IFNγ, TNFα, granzyme B in $CD8^+$ T cells with *HIF1A* overexpression. Data are presented as mean ± SD of samples from 4 donors. *P* values were determined by two-way ANOVA with Turkey's test. **c** Viability of human T cells with indicated treatments. Data are presented as the readings normalized to Day 0 and are expressed as the mean ± SD of three technical replicates. **d** Flow cytometric quantifications of TIM-3 and TIGIT in $CD8^+$ T cells gated from human pan-T cells with *HIF1A* knockdown. Data are presented as the mean ± SD of three independent experiments (n = 3). *P* values were determined by two-way ANOVA with Turkey's test. **e** Flow cytometric quantifications of TIM-3 and TIGIT in CD8+ T cells gated from human pan-T cells with indicated treatments. Data are presented as mean ± SD of samples from 3 donors. *P* values were determined by two-way ANOVA with Turkey's test. **f** Representative flow cytograms of PD-1 and TIM-3 expression in CD8 T cells with indicated treatment. **g** Flow cytometric quantifications of IFNγ in $PD\text{-}1^-TIM\text{-}3^-$, $PD\text{-}1^+TIM\text{-}3^-$, $PD\text{-}1^+TIM\text{-}3^+$ population of CD8 T cells with indicated treatments. Data are presented as mean ± SD of samples from 3 donors. *P* values were determined by two-way ANOVA with Turkey's test. Source data are provided as a Source Data file.

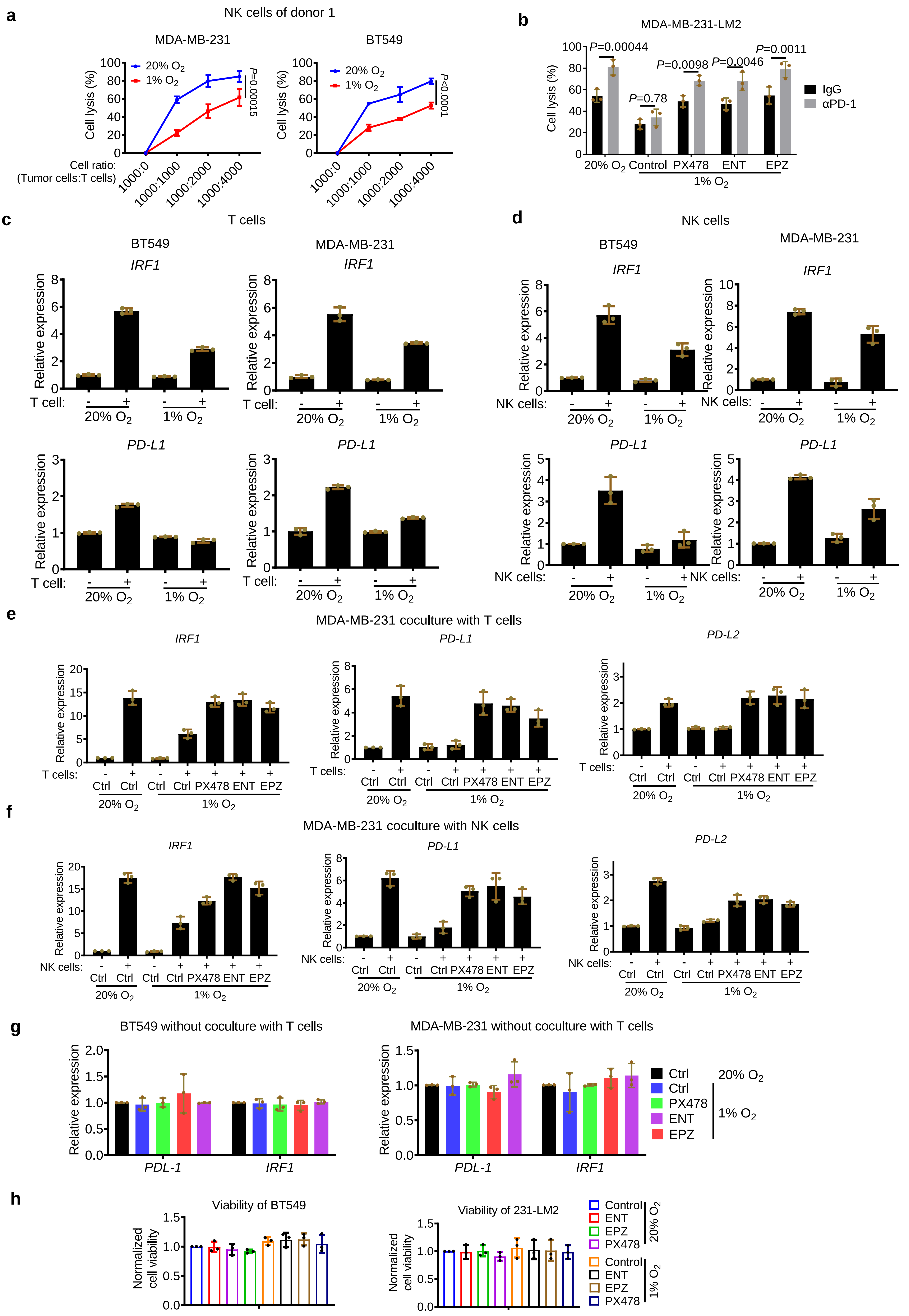

a
NK cells of donor 1
MDA-MB-231
BT549
Cell lysis (%)
20% O2
1% O2
P=0.00015
P<0.0001
Cell ratio: (Tumor cells:T cells)
1000:0
1000:1000
1000:2000
1000:4000
b
MDA-MB-231-LM2
P=0.00044
P=0.78
P=0.0098
P=0.0046
P=0.0011
IgG
αPD-1
20% O2
Control
PX478
ENT
EPZ
1% O2
c
T cells
d
NK cells
IRF1
PD-L1
Relative expression
T cell:
NK cells:
e
MDA-MB-231 coculture with T cells
PD-L2
T cells:
Ctrl
f
MDA-MB-231 coculture with NK cells
g
BT549 without coculture with T cells
MDA-MB-231 without coculture with T cells
PDL-1
h
Viability of BT549
Viability of 231-LM2
Normalized cell viability

Supplementary Figure 7. Treatment of human T or NK cells with inhibitors of HIF1α, HDAC1 or EZH2 rescues IFNγ-responsive signaling in TNBC cells under hypoxia. **a** Cell lysis of TNBC cells cocultured with human NK cells. Data are presented as mean ± SD of three independent experiments (n=3). *P* values were determined by two-way ANOVA. **b** Cell lysis of MDA-MB-231-LM2 cells cocultured with human T cells. Data are presented as mean ± SD of three independent experiments (n = 3). *P* values were determined by two-way ANOVA with Dunnett's test. **c-d** RT-qPCR analysis of *IRF1* and *PD-L1* in TNBC cells co-cultured with human T cells (**c**) or NK cells (**d**). **e-f** RT-qPCR analysis of *IRF1*, *PD-L1* and *PD-L2* in TNBC cells co-cultured with human T cells (**e**) or NK cells (**f**) with indicated treatments. **g** RT-qPCR analysis of *IRF1* and *PD-L1* in TNBC cells in monoculture. All qPCR data (**c-g**) are presented as the fold change of mRNA level normalized to control group (TNBC cells without T cells, under normoxia), mean ± SD of technical triplicates, representative of two independent experiments (n = 2). **h** Viability of BT549 and MDA-MB-231-LM2 cells with indicated treatments for 4 days. Data are presented as mean ± SD of three independent experiments (n = 3). *P* values were determined by two-way ANOVA. No significance was detected among groups (*P* >0.05). Source data are provided as a Source Data file.

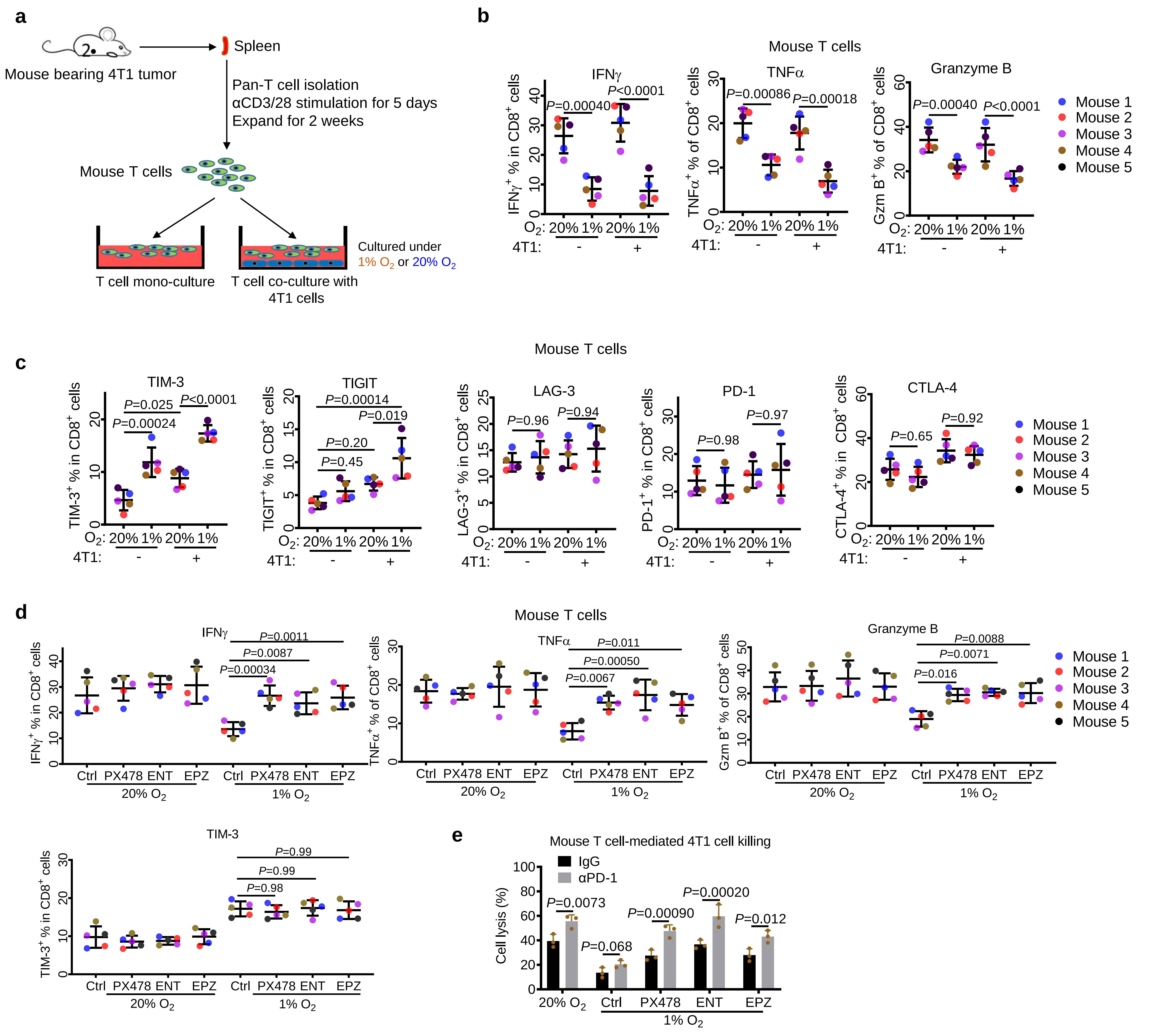

Supplementary Figure 8. Hypoxia induces dysfunction and impairs cytotoxicity of mouse T cells. **a** Schematic graph to demonstrate the isolation of mouse pan-T cells from the spleen of BALB/c mouse bearing 4T1 tumors and co-culture with 4T1 cells *in vitro*. **b** Flow cytometric quantifications of immune effector molecules in CD8^{+} T cells gated from mouse pan-T cells cultured under the indicated conditions. **c** Flow cytometric quantifications of exhaustion markers in CD8^{+} T cells gated from mouse pan-T cells cultured under the indicated conditions. **d** Flow cytometric quantifications of IFNγ, TNFα, granzyme B and TIM-3 in CD8^{+} T cells gated from mouse pan-T cells cultured under the indicated conditions. All flow cytometry data (**b**, **c** and **d**) are presented as the mean ± SD of samples from 5 mice. *P* values were determined by two-way ANOVA with Turkey's test. **e** Cell lysis of 4T1 cells co-cultured with mouse T cells. Data are presented as mean ± SD of three independent experiments (n = 3) using T cells from same mouse. *P* values were determined by two-way ANOVA. Source data are provided as a Source Data file.

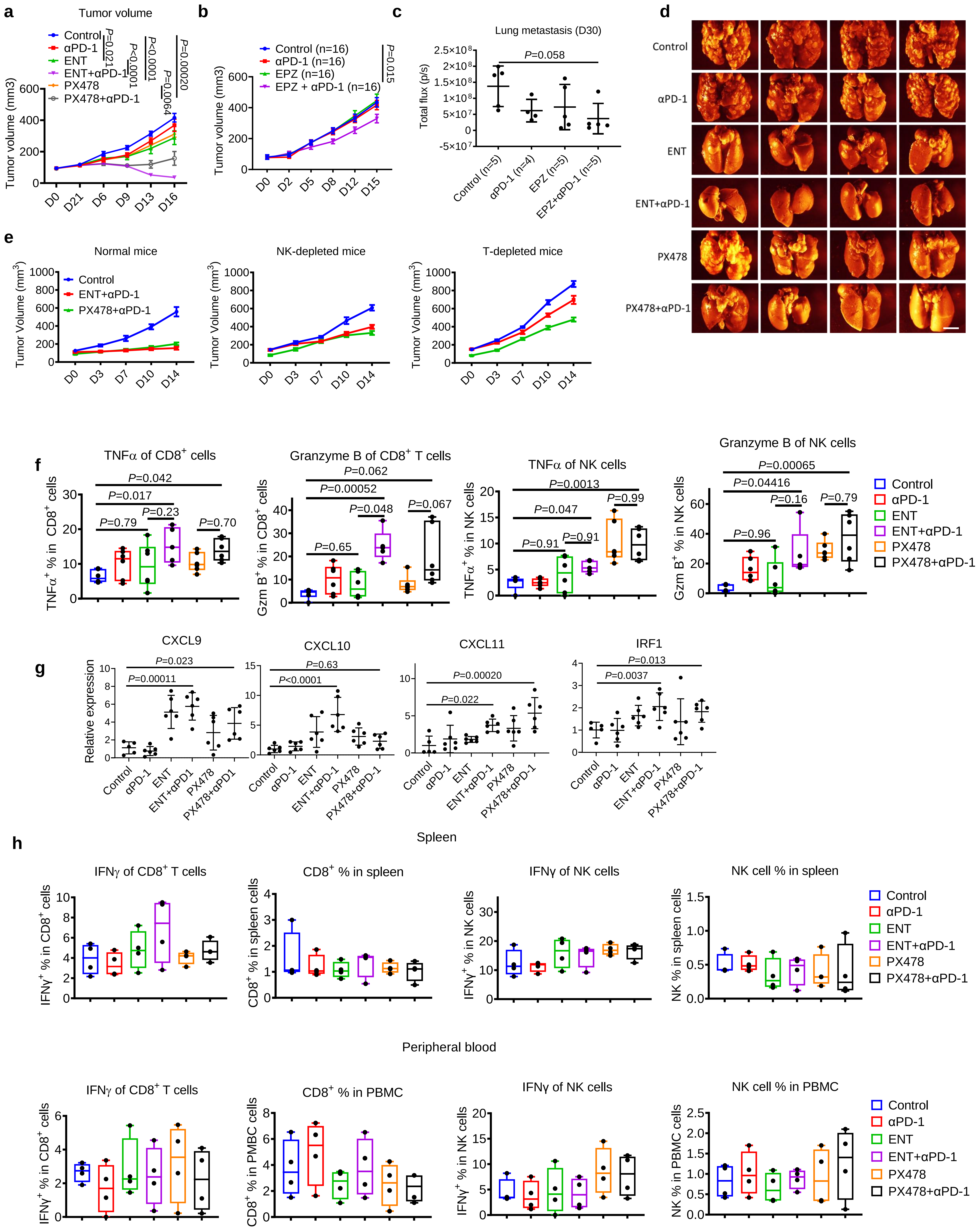

a
Tumor volume
Control
αPD-1
ENT
ENT+αPD-1
PX478
PX478+αPD-1
P=0.021
P<0.0001
P<0.0001
P=0.0064
P=0.00020
Tumor volume (mm3)
D0
D21
D6
D9
D13
D16
b
Control (n=16)
αPD-1 (n=16)
EPZ (n=16)
EPZ + αPD-1 (n=16)
P=0.015
Tumor volume (mm3)
D0
D2
D5
D8
D12
D15
c
Lung metastasis (D30)
P=0.058
Total flux (p/s)
Control (n=5)
αPD-1 (n=4)
EPZ (n=5)
EPZ+αPD-1 (n=5)
d
Control
αPD-1
ENT
ENT+αPD-1
PX478
PX478+αPD-1
e
Normal mice
NK-depleted mice
T-depleted mice
Control
ENT+αPD-1
PX478+αPD-1
Tumor Volume (mm3)
D0
D3
D7
D10
D14
f
TNFα of CD8+ cells
P=0.042
P=0.017
P=0.23
P=0.79
P=0.70
TNFα+ % in CD8+ cells
Granzyme B of CD8+ T cells
P=0.062
P=0.00052
P=0.048
P=0.067
P=0.65
Gzm B+ % in CD8+ cells
TNFα of NK cells
P=0.0013
P=0.99
P=0.047
P=0.91
P=0.91
TNFα+ % in NK cells
Granzyme B of NK cells
P=0.00065
P=0.04416
P=0.16
P=0.79
P=0.96
Gzm B+ % in NK cells
Control
αPD-1
ENT
ENT+αPD-1
PX478
PX478+αPD-1
g
CXCL9
P=0.023
P=0.00011
Relative expression
CXCL10
P=0.63
P<0.0001
CXCL11
P=0.00020
P=0.022
IRF1
P=0.013
P=0.0037
Control
αPD-1
ENT
ENT+αPD1
PX478
PX478+αPD1
h
Spleen
IFNγ of CD8+ T cells
IFNγ+ % in CD8+ cells
CD8+ % in spleen
CD8+ % in spleen cells
IFNγ of NK cells
IFNγ+ % in NK cells
NK cell % in spleen
NK % in spleen cells
Control
αPD-1
ENT
ENT+αPD-1
PX478
PX478+αPD-1
Peripheral blood
IFNγ of CD8+ T cells
IFNγ+ % in CD8+ cells
CD8+ % in PBMC
CD8+ % in PMBC cells
IFNγ of NK cells
IFNγ+ % in NK cells
NK cell % in PBMC
NK % in PBMC cells
Control
αPD-1
ENT
ENT+αPD-1
PX478
PX478+αPD-1

Supplementary Figure 9. PX478 and Entinostat sensitizes mouse 4T1 tumor to PD-1 blockade. **a** 4T1 tumor growth curve of BALB/c mice with indicated treatments. Data are presented as mean ± SD. N = 8. *P* values were determined by two-way ANOVA with Turkey's test. **b** 4T1 tumor growth curve of BALB/c mice. Data are presented as mean ± SD. N = 16. *P* values were determined by two-way ANOVA with Turkey's test. **c** Lung metastasis of BALB/c mice bearing 4T1 at Day 30. Data are presented as mean ± SD. N = 4 for αPD-1; n=5 for other groups. *P* values were determined by one-way ANOVA with Turkey's test. **d** Whole lung images of mice showing the metastatic nodules. Lung samples from Day 30 of treatment were stained with Bouin's solution. Scale bar, 5mm. Data is representative of 2 independent experiments. **e** 4T1 tumor growth curve of normal mice (control, n = 16; ENT+αPD-1, n = 14; PX478+αPD-1, n =10), T-depleted mice (n = 16 for control and ENT+αPD-1; PX478+αPD-1, n = 12) and NK-depleted mice (n = 16 for control and ENT+αPD-1; PX478+αPD-1, n = 10), NK cell-depleted and T cell-depleted mice with indicated treatments. **f** Flow cytometric analysis of 4T1 tumors harvested from mice with the indicated treatments. N = 6 for each group. **g** RT-qPCR analysis assessing expression of *CXCL9/10/11* and *IRF1* in 4T1 tumor samples with indicated treatments. Data are presented as mean ± SD, calculated as the fold change of mRNA level normalized to the mean value of control group. (N=5 for control group; n=6 for other groups). *P* values were determined by one-way ANOVA with Dunnett's test. **h** Flow cytometric analysis of spleen and PBMC harvested from mice bearing 4T1 tumors. N = 5 for each group. Flow cytometric data (**f** and **h**) are presented as as box and whiskers, with median value and whiskers of minimum and maximum values. For flow cytometric data (**f** and **h**), *P* values were determined by one-way ANOVA with Turkey's test. No significance was observed between any two groups in **h** (*P* > 0.05). Source data are provided as a Source Data file.

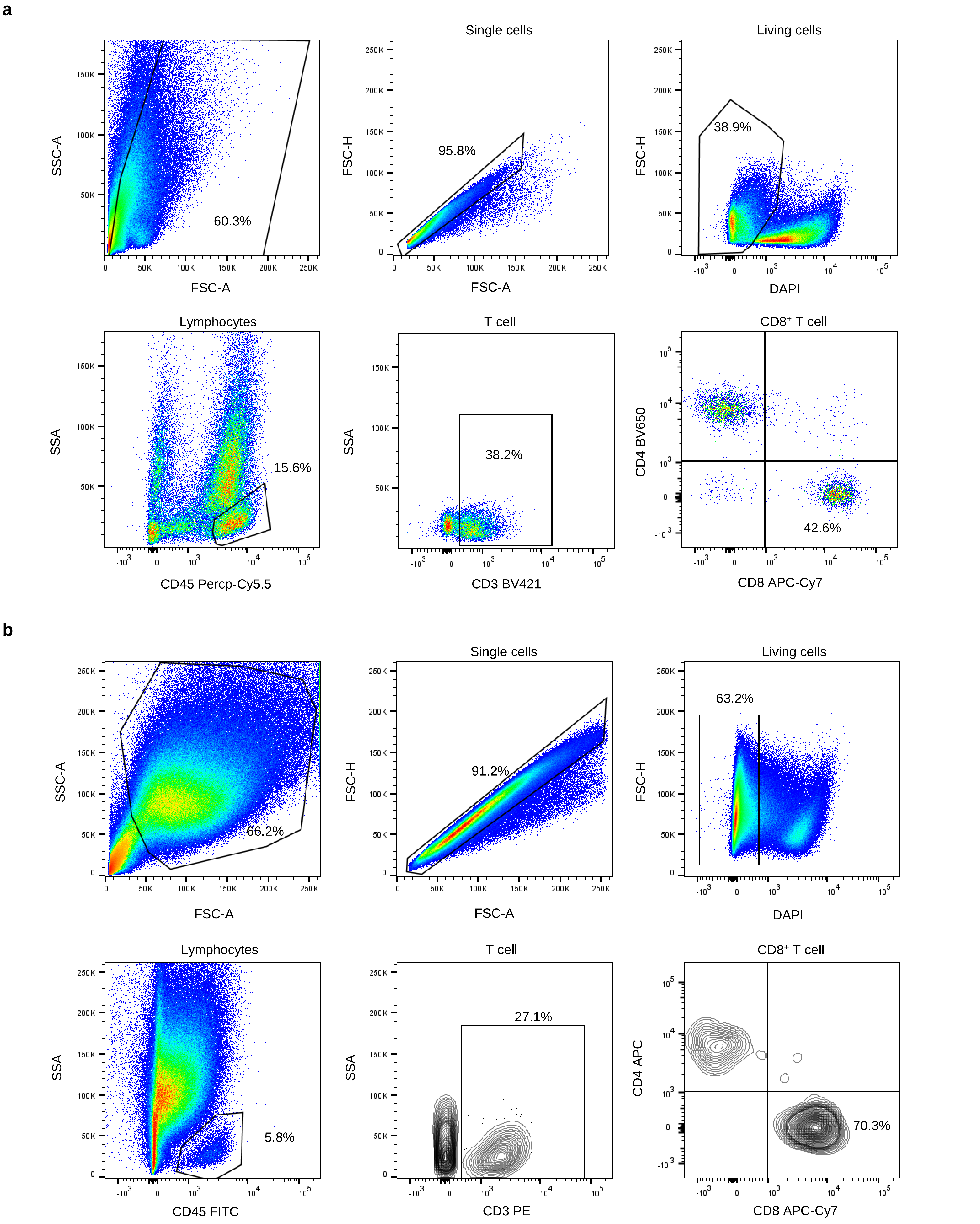

Supplementary Figure 10. Flow cytometry gating strategy. Representative flow cytograms to demonstrate gating strategies for BALB/c 4T1 tumors (**a**) and humanized LM2 tumors (**b**)

Supplementary table 1. RT-qPCR primer list

Human gene		Sequence
GZMB	Forward	GATCATCGGGGGACATGAGG
	Reverse	TGACATTTATGGAGCTTCCCCA
TNF	Forward	GCCCATGTTGTAGCAAACCC
	Reverse	GGTTATCTCTCAGCTCCACGC
IFNG	Forward	AGTGATGGCTGAACTGTCGC
	Reverse	CTCTTCGACCTCGAAACAGC
PDL2	Forward	ACCCTGGAATGCAACTTTGAC
	Reverse	AAGTGGCTCTTTCACGGTGTG
PDL1	Forward	GTTGAAGGACCAGCTCTCCC
	Reverse	CTTGTAGTCGGCACCACCAT
IRF1	Forward	GAGGAGGTGAAAGACCAGAGCA
	Reverse	TAGCATCTCGGCTGGACTTCGA
GAPDH	Forward	TGCACCACCAACTGCTTAGC
	Reverse	GGCATGGACTGTGGTCATGAG
Mouse gene		Sequence
GAPDH	Forward	CATCACTGCCACCCAGAAGACTG
	Reverse	ATGCCAGTGAGCTTCCCGTTCAG
IRF1	Forward	GGCCGATACAAAGCAGGAGAA
	Reverse	GGAGTTCATGGCACAACGGA
CXCL9	Forward	GGAGTTCGAGGAACCCTAGTG
	Reverse	GGGATTTGTAGTGGATCGTGC
CXCL10	Forward	CCAAGTGCTGCCGTCATTTTC
	Reverse	TCCCTATGGCCCTCATTCTCA
CXCL11	Forward	CCGAGTAACGGCTGCGACAAAG
	Reverse	CCTGCATTATGAGGCGAGCTTG

Supplementary table 2. ChIP-qPCR primer list

Human gene		
IFNG promoter region1 (P1)	Forward	TCAAGTTTGCCCCATAACTGC
	Reverse	GCACAAAAAGCCCTCCACTC
IFNG promoter region2 (P2)	Forward	CCGCATTCTTTCCTTGCTTTCT
	Reverse	AGGGCCTCTCAAACCTTTACAA
IFNG promoter region3 (P3)	Forward	GGTGGGCATAATGGGTCTGT
	Reverse	GGTTTTGTGGCATTTGGGTGT
IFNG promoter region4 (P4)	Forward	TGGCTTAATTCTCTCGGAAACG
	Reverse	ATGGGTCCTGGCAGTAACAG
TNF promoter region1 (P1)	Forward	GAGGGACAGAGGGCTCAAAG
	Reverse	CACCTGGTGAGTCCATCACAT
TNF promoter region2 (P2)	Forward	AAGATATGGCCACACACTGG
	Reverse	TCTGACCCGGAGACTCATAAT
TNF promoter region3 (P3)	Forward	CGCCACATCCCCTGACA
	Reverse	CGTGGGTCAGTATGTGAGAGGAA
TNF promoter region4 (P4)	Forward	AGAGCTGTTGAATGCCTGGAA
	Reverse	CTGGCCTGCGCTCTTAGC
CCND2	Forward	TAGGATCCGTTTTGAAGAAGCC
	Reverse	CATTCTGTAGGTGTAGCACGCC
VEGFA	Forward	GCCTCTGTCTGCCCAGCTGC
	Reverse	GTG GAGCTGAGAACGGGAAGC

Supplementary table 3. List of sgRNA for CRISPR-based gene knockdown

Target genes		
HIF1A	sgRNA #1	TGTTTACAGTTTGAACTAAC
	sgRNA #2	TACTCATCCATGTGACCATG
HDAC1	sgRNA #1	ATTGACATTGATATTCACCA
	sgRNA #2	CTGGATACGGAGATCCCTAA
EZH2	sgRNA #1	GGTCCCAATTAACCTAGCAA
	sgRNA #2	CAACACCCAACACTTATAAG
